# Supplementary material for: Pre-existing technological core and roots for the CRISPR breakthrough
Source: PLoS One. 2018 Sep 19;13(9):e0198541. doi: 10.1371/journal.pone.0198541 (PMC6145527; doi:10.1371/journal.pone.0198541)
Supplement: S1 Table — Included are the patent number, title, publication year, inventors and assignees for each patent. (PDF) [file pone.0198541.s001.pdf]

The table below lists the 58 patents in genome engineering used as a pre-set for finding relevant UPC and CPC classes representing genome engineering.

Category	Patent Number	Title	Publication Year	Inventor(s)	Assignee(s)
ZFN	US6479626	Poly zinc finger proteins with improved linkers	2002	Kim, Jin-Soo | Pabo, Carl O.	Massachusetts Institute of Technology
ZFN	US6503717	Methods of using randomized libraries of zinc finger proteins for the identification of gene function	2003	Case, Casey C. | Liu, Qiang | Rebar, Edward J. | Wolffe, Alan P.	Sangamo Biosciences, Inc.
ZFN	US6534261	Regulation of endogenous gene expression in cells using zinc finger proteins	2003	Cox, Iii, George Norbert | Case, Casey Christopher | Eisenberg, Stephen P. | Jarvis, Eric Edward | Spratt, Sharon Kaye	Sangamo Biosciences, Inc.
ZFN	US6599692	Functional genomics using zinc finger proteins	2003	Case, Casey C. | Zhang, Lei	Sangamo Bioscience, Inc.
ZFN	US6607882	Regulation of endogenous gene expression in cells using zinc finger proteins	2003	Cox, Iii, George N. | Case, Casey C. | Eisenberg, Stephen P. | Jarvis, Eric E. | Spratt, Sharon K.	Sangamo Biosciences, Inc.
ZFN	US6733970	Screening system for zinc finger polypeptides for a desired binding ability	2004	Choo, Yen | Moore, Michael	Gendaq Limited

ZFN	US6746838	Nucleic acid binding proteins	2004	Choo, Yen | Klug, Aaron | Isalan, Mark	Gendaq Limited
ZFN	US6777185	Functional genomics using zinc finger proteins	2004	Case, Casey C. | Zhang, Lei | Urnov, Fyodor	Sangamo Biosciences, Inc.
ZFN	US6785613	Selection of sites for targeting by zinc finger proteins and methods of designing zinc finger proteins to bind to preselected sites	2004	Eisenberg, Stephen P. | Case, Casey C. | Cox, Iii, George N. | Jamieson, Andrew | Rebar, Edward J.	Sangamo Biosciences, Inc.
ZFN	US6794136	Iterative optimization in the design of binding proteins	2004	Eisenberg, Stephen P. | Liu, Qiang | Jamieson, Andrew | Rebar, Edward	Sangamo Biosciences, Inc.
ZFN	US6824978	Regulation of endogenous gene expression in cells using zinc finger proteins	2004	Cox, Iii, George Norbert | Case, Casey Christopher | Eisenberg, Stephen P. | Jarvis, Eric Edward | Spratt, Sharon Kaye	Sangamo Biosciences, Inc.
ZFN	US6866997	Nucleic acid binding proteins	2005	Choo, Yen | Klug, Aaron | Isalan, Mark	Gendaq Limited
ZFN	US6903185	Poly zinc finger proteins with improved linkers	2005	Kim, Jin-Soo | Pabo, Carl O.	Massachusetts Institute of Technology

ZFN	US6933113	Modulation of endogenous gene expression in cells	2005	Case, Casey C. | Wolffe, Alan | Urnov, Fyodor | Lai, Albert | Snowden, Andrew | Tan, Siyuan | Gregory, Philip	Sangamo Biosciences, Inc.
ZFN	US6979539	Regulation of endogenous gene expression in cells using zinc finger proteins	2005	Cox, Iii, George Norbert | Case, Casey Christopher | Eisenberg, Stephen P. | Jarvis, Eric Edward | Spratt, Sharon Kaye	Sangamo Biosciences, Inc.
ZFN	US7013219	Regulation of endogenous gene expression in cells using zinc finger proteins	2006	Case, Casey C. | Cox, Iii, George N. | Eisenberg, Stephen P. | Liu, Qiang | Rebar, Edward J.	Sangamo Biosciences, Inc.
ZFN	US7030215	Position dependent recognition of GNN nucleotide triplets by zinc fingers	2006	Liu, Qiang | Rebar, Edward | Jamieson, Andrew C.	Sangamo Biosciences, Inc.
ZFN	US7220719	Modulation of endogenous gene expression in cells	2007	Case, Casey C. | Wolffe, Legal Representative, Elizabeth | Urnov, Fyodor | Lai, Albert | Snowden, Andrew | Tan, Siyuan | Gregory, Philip | Wolffe, Deceased, Alan	Sangamo Biosciences, Inc.
ZFN	US7241573	Nucleic acid binding proteins	2007	Choo, Yen | Klug, Aaron | Isalan, Mark	Gendaq Ltd.
ZFN	US7241574	Nucleic acid binding proteins	2007	Choo, Yen | Klug, Aaron | Isalan, Mark	Gendaq Ltd.

ZFN	US7585849	Position dependent recognition of GNN nucleotide triplets by zinc fingers	2009	Liu, Qiang | Rebar, Edward | Jamieson, Andrew C.	Sangamo Biosciences, Inc.
ZFN	US7595376	Poly zinc finger proteins with improved linkers	2009	Kim, Jin-Soo | Pabo, Carl O.	Massachusetts Institute of Technology
Meganuclease	US7842489	Use of meganucleases for inducing homologous recombination ex vivo and in toto in vertebrate somatic tissues and application thereof	2010	Arnould, Sylvain | Bruneau, Sylvia | Cabaniols, Jean-Pierre | Chames, Patrick | Choulika, Andre | Duchateau, Philippe | Epinat, Jean-Charles | Gouble, Agnes | Lacroix, Emmanuel | Paques, Frederic | Perez-Michaut, Christophe | Smith, Julianne | Sourdive, David	Cellectis
ZFN	US7888121	Methods and compositions for targeted cleavage and recombination	2011	Urnov, Fyodor | Holmes, Michael C. | Miller, Jeffrey C. | Pabo, Carl O.	Sangamo Biosciences, Inc.
Meganuclease	US8021867	Rationally-designed meganucleases with altered sequence specificity and DNA-binding affinity	2011	Smith, James J. | Jantz, Derek | Hellinga, Homme W.	Duke University
Meganuclease	US8119361	Methods of cleaving DNA with rationally-designed meganucleases	2012	Smith, James Jefferson | Jantz, Derek | Hellinga, Homme W.	Duke University

Meganuclease	US8119381	Rationally-designed meganucleases with altered sequence specificity and DNA-binding affinity	2012	Smith, James Jefferson | Jantz, Derek | Hellinga, Homme W.	Duke University
Meganuclease	US8163514	Methods of cleaving DNA with rationally-designed meganucleases	2012	Smith, James Jefferson | Jantz, Derek | Hellinga, Homme W.	Duke University
TALEN	US8420782	Modular DNA-binding domains and methods of use	2013	Bonas, Ulla | Boch, Jens | Schornack, Sebastian | Lahaye, Thomas	Bonas, Ulla | Boch, Jens | Schornack, Sebastian | Lahaye, Thomas
TALEN	US8440431	TAL effector-mediated DNA modification	2013	Voytas, Daniel F. | Bogdanove, Adam J. | Zhang, Feng	Regents of The University of Minnesota | Iowa State University Research Foundation, Inc.
TALEN	US8440432	Tal effector-mediated DNA modification	2013	Voytas, Daniel F. | Bogdanove, Adam J. | Zhang, Feng	Regents of The University of Minnesota | Iowa State University Research Foundation, Inc.
TALEN	US8450471	TAL effector-mediated DNA modification	2013	Voytas, Daniel F. | Bogdanove, Adam J. | Zhang, Feng	Regents of The University of Minnesota | Iowa State University Research Foundation, Inc.
CRISPR	US8697359	CRISPR-Cas systems and methods for altering expression of gene products	2014	Zhang, Feng	The Broad Institute, Inc. | Massachusetts Institute of Technology

TALEN	US8697853	TAL effector-mediated DNA modification	2014	Voytas, Daniel F. | Bogdanove, Adam J. | Zhang, Feng	Regents of The University of Minnesota | Iowa State University Research Foundation, Inc.
CRISPR	US8771945	CRISPR-Cas systems and methods for altering expression of gene products	2014	Zhang, Feng	The Broad Institute, Inc. | Massachusetts Institute of Technology
ZFN	US8802921	Engineered landing pads for gene targeting in plants	2014	Ainley, William M. | Blue, Ryan C. | Murray, Michael G. | Corbin, David | Miles, Rebecca R. | Webb, Steven R.	Dow Agrosciences, Llc.
CRISPR	US8865406	Engineering and optimization of improved systems, methods and enzyme compositions for sequence manipulation	2014	Zhang, Feng | Ran, Fei	The Broad Institute, Inc. | Massachusetts Institute of Technology
CRISPR	US8889418	Engineering and optimization of improved systems, methods and enzyme compositions for sequence manipulation	2014	Zhang, Feng | Ran, Fei	The Broad Institute, Inc. | Massachusetts Institute of Technology
CRISPR	US8895308	Engineering and optimization of improved systems, methods and enzyme compositions for sequence manipulation	2014	Zhang, Feng | Ran, Fei	The Broad Institute, Inc. | Massachusetts Institute of Technology
Meganuclease	US8906607	Method for modulating double-strand break-induced homologous recombination	2014	Duchateau, Philippe | Paques, Frederic | Perez-Michaut, Christophe | Delacote, Fabien	Cellectis

ZFN	US8921112	Optimized non-canonical zinc finger proteins	2014	Cai, Qihua C. | Shukla, Vipula K. | Petolino, Joseph F. | Baker, Lisa W. | Garrison, Robbi J. | Blue, Ryan C. | Mitchell, Jon C. | Arnold, Nicole L. | Worden, Sarah E. | Miller, Jeffrey | Urnov, Fyodor	Dow Agrosciences Llc | Sangamo Biosciences, Inc.
ZFN / Meganuclease	US8921332	Chromosomal modification involving the induction of double-stranded DNA cleavage and homologous recombination at the cleavage site	2014	Choulika, Andre | Mulligan, Richard C.	Children'S Medical Center Corporation | Institut Pasteur
CRISPR	US8932814	CRISPR-Cas nickase systems, methods and compositions for sequence manipulation in eukaryotes	2015	Cong, Le | Zhang, Feng	The Broad Institute, Inc. | Massachusetts Institute of Technology
CRISPR	US8945839	CRISPR-Cas systems and methods for altering expression of gene products	2015	Zhang, Feng	The Broad Institute, Inc. | Massachusetts Institute of Technology
CRISPR	US8993233	Engineering and optimization of systems, methods and compositions for sequence manipulation with functional domains	2015	Zhang, Feng | Cong, Le | Platt, Randall Jeffrey | Sanjana, Neville Espi | Ran, Fei	The Broad Institute Inc. | Massachusetts Institute of Technology | President and Fellows of Harvard College
CRISPR	US8999641	Engineering and optimization of systems, methods and compositions for sequence manipulation with functional domains	2015	Zhang, Feng | Cong, Le | Platt, Randall Jeffrey | Sanjana, Neville Espi	The Broad Institute Inc. | Maassachusetts Institute of Technology | President and Fellows of Harvard College

CRISPR	US9023649	RNA-guided human genome engineering	2015	Mali, Prashant G. | Church, George M. | Yang, Luhan	President and Fellows of Harvard College
CRISPR	US9074199	Mutant Cas9 proteins	2015	Chavez, Alejandro | Poelwijk, Frank | Church, George M.	President and Fellows of Harvard College
ZFN	US9187758	Optimized non-canonical zinc finger proteins	2015	Cai, Qihua C. | Shukla, Vipula K. | Petolino, Joseph F. | Baker, Lisa W. | Garrison, Robbi J. | Blue, Ryan C. | Mitchell, Jon C. | Arnold, Nicole L. | Worden, Sarah E. | Miller, Jeffrey | Urnov, Fyodor	Sangamo Biosciences, Inc. | Dow Agrosciences Llc
CRISPR	US9260723	RNA-guided human genome engineering	2016	Mali, Prashant G. | Church, George M. | Yang, Luhan	President And Fellows of Harvard College
CRISPR	US9260752	Compositions and methods of nucleic acid-targeting nucleic acids	2016	May, Andrew Paul | Haurwitz, Rachel E. | Doudna, Jennifer A. | Berger, James M. | Carter, Matthew Merrill | Donohoue, Paul	Caribou Biosciences, Inc.
CRISPR	US9267135	RNA-guided transcriptional regulation	2016	Church, George M. | Mali, Prashant G. | Esvelt, Kevin M.	President and Fellows of Harvard College

TALEN	US9315788	Method for the generation of compact TALE-nucleases and uses thereof	2016	Duchateau, Philippe | Valton, Julien | Bertonati, Claudia | Epinat, Jean-Charles | Silva, George H. | Juillerat, Alexandre | Beurdeley, Marine	Cellectis, S.A.
TALEN	US9393257	TALEN-based gene correction	2016	Osborn, Mark J. | Tolar, Jakub | Blazar, Bruce | Voytas, Daniel F.	Regents of The University of Minnesota
CRISPR	US9410198	Compostions and methods of nucleic acid-targeting nucleic acids	2016	May, Andrew Paul | Haurwitz, Rachel E. | Doudna, Jennifer A. | Berger, James M. | Carter, Matthew Merrill | Donohoue, Paul	Caribou Biosciences, Inc.
TALEN	US9499592	Transcription activator-like effectors	2016	Zhang, Feng | Cong, Le | Kosuri, Sriram | Church, George M.	President And Fellows of Harvard College
CRISPR	US9587252	Orthogonal Cas9 proteins for RNA-guided gene regulation and editing	2017	Church, George M. | Esvelt, Kevin | Mali, Prashant	President and Fellows of Harvard College
ZFN	US9695432	Excision of transgenes in genetically modified organisms	2017	Russell, Sean M. | Petolino, Joseph F.	Dow Agrosciences Llc
